# Influence of palmitoylation in axonal transport mechanisms in neurodegenerative diseases

**DOI:** 10.3389/fncel.2025.1613379

**Published:** 2025-08-04

**Authors:** Praveen B. S., Priti Talwar

**Affiliations:** Apoptosis and Cell Survival Research Laboratory, School of Biosciences and Technology, Vellore Institute of Technology, Vellore, India

**Keywords:** axonal transport, palmitoylation, depalmitoylation, zDHHC, neurodegenerative diseases

## Abstract

Progressive functional loss and death of neurons are characteristics of neurodegenerative diseases such as Alzheimer’s disease (AD), Amyotrophic lateral sclerosis (ALS), and Parkinson’s disease (PD). These diseases are often linked with disruptions in axonal transport and synaptic functions. Accumulation of misfolded proteins is observed as a commonly shared pathology for these diseases, where aberrant accumulation of amyloid beta (Aβ), tau, α-synuclein (α-syn) and TAR DNA-binding protein 43 (TDP-43), are found in AD, PD and ALS, respectively. These accumulations are observed to be involved in disrupting axonal transport and compromising neuronal survival. Axonal transport is an essential process where proper functioning of the transport mechanism is important for maintaining neuronal hemostasis by transporting of proteins, organelles and neurotransmitter complexes. This review explores the role of palmitoylation in regulating neuronal axonal transport and their impact on other neuronal functions along with neurodegeneration mechanisms. Palmitoylation is a reversible lipid modification, which is widely studied second to phosphorylation. Enzymes like palmitoyl acyltransferases and acyl-protein thioesterases are responsible for attachment and detachment of palmitic acid causing palmitoylation and depalmitoylation of neuronal proteins. In axonal transport, palmitoylation influences the localization and functioning of the proteins, which connectively plays a role in synaptic stability by interacting with synaptic scaffolding proteins and neurotransmission receptors.

## 1 Introduction

Progressive functional loss and death of neurons is known as a hallmark of neurodegenerative diseases like Alzheimer’s disease (AD), Amyotrophic lateral sclerosis (ALS) and Parkinson’s disease (PD). The functional disruption of intracellular transport and synaptic networks that result in a build-up of accumulation of misfolded proteins like amyloid beta (Aβ), tau, α-synuclein (α-syn) and TAR DNA-binding protein 43 (TDP-43) are found in AD, PD and ALS, respectively. Disruption of axonal transport is observed as a commonality between these diseases, which is also the reason for aberrant accumulation of such misfolded proteins affecting the communication and survival of the neurons (Wilson et al., 2023; Forrest and Kovacs, 2024). In axonal transport, the components necessary for proper neuronal functioning, such as proteins, organelles and neurotransmission complexes, are transported on an intermediate filamentous scaffolded protein called microtubule. The transport mechanism on the microtubule is mediated by two main proteins: kinesin (anterograde transport toward the synapse) and dynein (retrograde transport toward the cell body). Kinesin and Dynein essentially facilitates the functioning of several cellular activities and disruption of this mechanism often exhibits synaptic loss, cognitive impairment, and neuronal death in several neurodegenerative conditions (De Vos et al., 2008; Hirokawa et al., 2010). Disruption of axonal transport causes synaptic impairment by restricting the accessibility of necessary neurotransmitters and organelles, which can affect the functional neuron interaction and additionally contribute to neuronal injury by microglial activation, neuroinflammation and releasing pro-inflammatory cytokines (Takeuchi et al., 2005; Hirokawa et al., 2010). In AD, accumulation of tau proteins causes instability of microtubules that consequentially impairs the axonal transport, thus leading to synaptic loss and cognitive impairment (Manczak and Reddy, 2012). Similarly, failure in the pace of the transport mechanism in ALS leads to motor neuron degeneration and in PD impairs the functions of dopaminergic neurons (Prots et al., 2018; Suzuki et al., 2020).

Among the varied cellular mechanisms influencing neuronal function, palmitoylation modification is the second widely studied post-translational modification (PTM) next to Phosphorylation. Palmitoylation (also called S-Acetylation) is a reversible modification that involves the attachment of palmitic acid to the cysteine amino acid of the protein, which effectively regulates the critical mechanism necessary for localizing and functioning of scaffolding and synaptic proteins. It is one of the vital processes involved in regulating critical activities like protein trafficking, signal transduction and synaptic stability. Palmitoylation dysregulation contributes to impairment of synaptic communication and axonal transport instability in neurodegenerative diseases (Ramzan et al., 2023). In AD, the involvement of abnormal modifications at the site of tau’s microtubule binding domain causes neurotoxicity by tau aggregation (García-Cruz and Arias, 2024). Similarly in PD, dysregulated modifications of α-synuclein accelerates the degeneration of dopaminergic neurons by affecting mitochondrial activity and vesicular transport (Ho et al., 2021). While previous study have discussed the role of palmitoylation in neurons and neurodegenerative diseases in general, this review focuses specifically on how palmitoylation regulates axonal transport. Here, we bring together recent findings that show how palmitoylation, especially through zDHHC enzymes, affects the movement and function of key transport proteins in neurons. We also highlight how changes in palmitoylation contribute to the transport defects seen in diseases such as AD, PD and ALS. By connecting these molecular mechanisms with disease outcomes, this review provides a focused perspective on how palmitoylation influences axonal transport and outlines how this connection could lead to new research directions and therapeutic strategies in neurodegenerative diseases.

## 2 Axonal transport components and their mechanism

### 2.1 Microtubules in axonal transport

**FIGURE 1:**
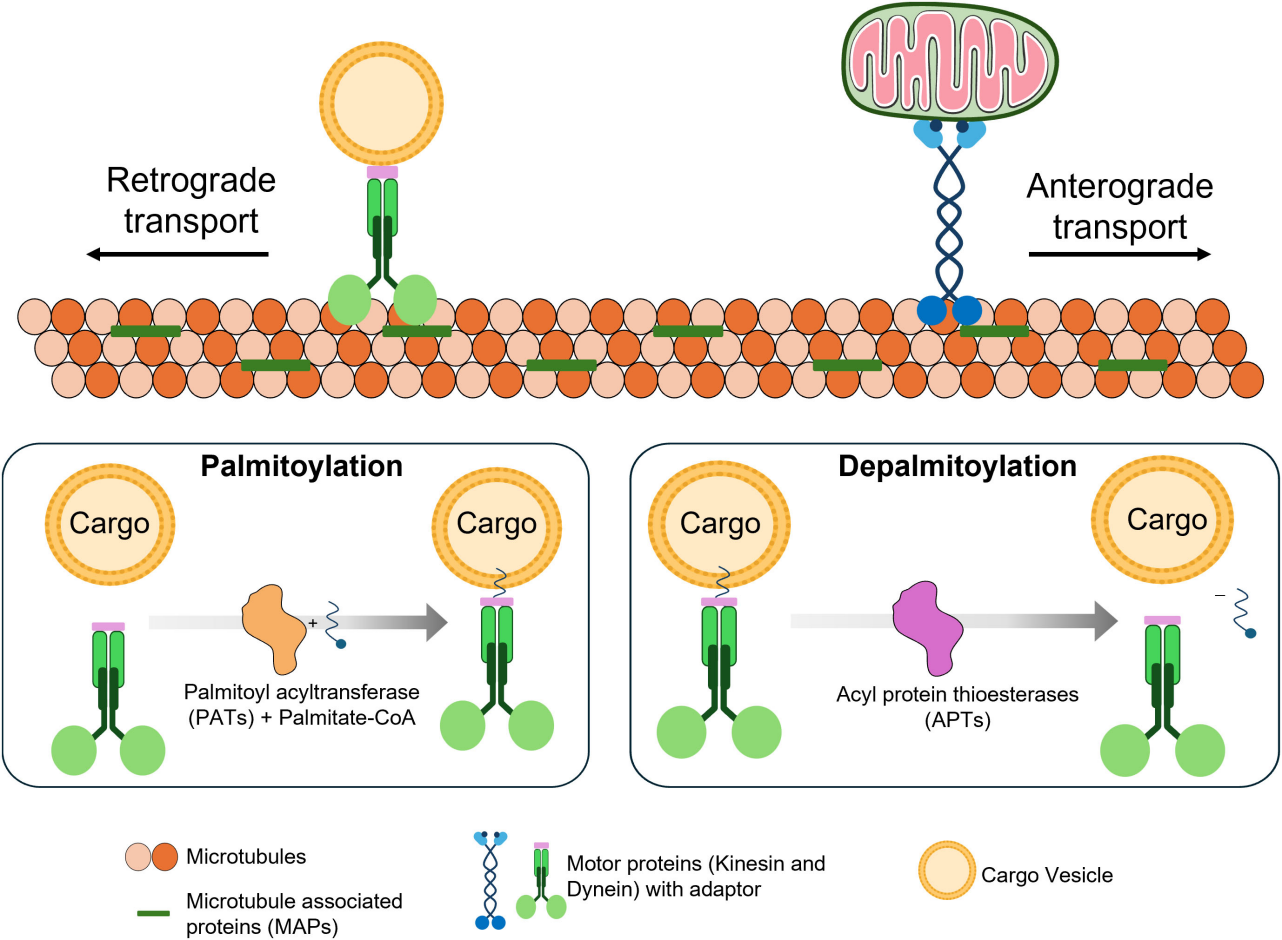
Role of palmitoylation in axonal transport. Microtubules are organized, stabilized and formed as a network by microtubule-associated proteins (MAPs). Along this network, motor proteins (kinesin and dynein) transports cargo using energy derived from ATP hydrolysis. These motor proteins move stepwise along microtubules in a directional manner, powered by the hydrolysis of ATP at each step upon interaction with microtubules. Cargoes are linked to motor proteins through adaptor proteins, which facilitate specific attachment. These adaptor proteins can undergo palmitoylation, a lipid modification that enhances their membrane association (by anchoring the adaptor to the cargo) and stabilizes their connection to the cargo. Upon depalmitoylation, the adaptor’s affinity for the membrane decreases, and then releases the cargo from the transportation complex.

Axonal transport is an essential mechanism that supports the proper physiological functioning of neurons by efficiently channeling the intracellular cargo trafficking (Huang et al., 2022). Axonal transport acts as a central network in neurons by facilitating essential cargoes to different parts of neurons, by aiding in vital processes like synaptic plasticity and neurotransmission. In neuron axonal transport, the core component, microtubules serve as a primary channel for intracellular cargo trafficking, where the structural organization of microtubules controls the efficiency of axonal transport. Microtubules are a well-organized array bundle and spatially aligned scaffold proteins that efficiently enable axonal transport’s pace and functioning (Stephan et al., 2015). Furthermore, other properties of microtubules, like their arrangement, polarity and stability, regulate the axonal transport efficacy and direction, resulting in adequate neuronal functioning and integrity. Disruptions in microtubule physiology affect the axonal transport mechanism and eventually cause neurodegenerative disease (Yogev et al., 2016; Costa and Sousa, 2022). Microtubules exhibit a polarized orientation, with the plus-end leading toward the axon terminal and the minus-end toward the cell body. This polarized orientation helps guide the motor protein directions, which facilitates kinesin and dynein with anterograde and retrograde transport directions, respectively. PTMs on microtubules like acetylation and tyrosination influence the efficiency of these motor proteins movements by regulating their affinity and sorting (Tas et al., 2017). Microtubule stability in axonal transport determines the motor protein recruitment and movement efficiency for cargo transport. By stabilizing microtubules, tubulin acetylation increases the ability to bind and retain the motor proteins, specifically for dynein in retrograde transport. Motor proteins like kinesin and dynein bind with the microtubule and other end of the motor proteins binds to the cargoes via adaptors, this interaction between the motor protein and cargo is facilitated by palmitoylation of adaptor proteins illustrated in Figure 1.

### 2.2 Motor proteins

Motor proteins like kinesin and dynein transport along the microtubule network effectively carrying the cargo movement are essential for axonal transport. Kinesin and dynein support the anterograde and retrograde transportation respectively, where the motor proteins that transports the cargo from the cell body to the axonal terminal at the synaptic terminals are called anterograde transportation, and the reverse is called retrograde transportation. These movements make it easier for kinesin and dynein to effectively transport necessary cargo like organelles, proteins, vesicles and cell signaling responses for processes like synaptic functions, recycling cell organelles, neuronal development and maintenance. Kinesin and dynein work coordinately in both directions, which upon disruption of one motor protein’s function tends to affect the other, indicating their connective interdependence (Encalada et al., 2011; Lee et al., 2011). Neurofilament transport is the paramount example of the concerted action of kinesin and dynein in axonal transport, where kinesin follows the anterograde movement, and dynein follows the retrograde movement. Their actions are tightly coupled to regulate efficient bi-directional transport. Kinesin-1 drives the transport of mitochondria to high energy demand regions like synaptic terminals and dynein drives the degradation and recycling of damaged or aged mitochondria toward the cell body (Pilling et al., 2006; Uchida et al., 2009; Twelvetrees et al., 2016). When impaired, the mutually dependent bi-directional movements of kinesin and dynein can lead to mitochondrial buildup, energy deficit, and neuronal deterioration, which are hallmarks of many neurodegenerative diseases (Wilson et al., 2023).

### 2.3 Microtubule-associated proteins (MAPs)

Additionally to motor proteins, MAPs are also essential to maintain the microtubules stability, axonal structure and transport efficiency by promoting microtubule bundling (Tortosa et al., 2013; Tas et al., 2017). MAPs are critically important in regulating microtubule stability and neuronal integrity, which influences axonal transport’s direction, organization and speed by interacting with microtubules and motor proteins. MAPs dysregulations can cause disruption in the transport mechanisms leading to synaptic dysfunction and neuron degeneration. The classic example of such a case is tau protein hyperphosphorylation followed by aggregation, an important pathological feature in neurodegenerative diseases. Tau is an essential MAP member that promotes axon elongation, microtubule stability and intracellular transport support. Tau, in a normal state, binds to the microtubules to stabilize them and acts as a scaffold for motor proteins to transport cargo. However, hyperphosphorylated and aggregated conditions of tauopathies, such as AD and frontotemporal dementia (FTD), impair the transport mechanism and destabilize microtubules (Iqbal et al., 2010; Alonso et al., 2018). The tangles formed by aggregated hyperphosphorylated proteins called neurofibrillary tangles (NFT), are a hallmark of AD. These tangles disrupt the anterograde and retrograde transport movement of motor proteins, causing an imbalance in the homeostasis of neuron and synaptic functions. This imbalance in the transport mechanism of tau proves to have differential effects on kinesin and dynein. It is observed that tau potentially affects anterograde transport more than retrograde transport by inhibiting Kinesin-1. This results in increased retrograde transport and accumulation of cargo in the axon, increasing the synaptic function impairment (Dixit et al., 2008; Chaudhary et al., 2018). Dynein-mediated transport is also affected by tau modifications. Although dynein is less susceptible to tau inhibition compared to kinesin, high tau levels can still impair dynein-dependent transport, particularly in early phagosomes, by lowering the interacted dynein motors numbers (Dixit et al., 2008; Vershinin et al., 2008). The characteristic features of tauopathies are synaptic dysfunction and neuronal degeneration; transport imbalance can result in both disturbances leading to tauopathies.

#### 2.3.1 Tau protein

Tau protein is encoded by microtubule-associated protein tau (MAPT) gene, which is one of the well-studied MAPs in the context of axonal transport. Tau proteins are an essential component for the regulation of stabilized microtubule networks. Hyperphosphorylation of tau disrupts these functions and is known as a key pathogenic hallmark of AD, where aggregated tau forms neurofibrillary tangles (NFTs). Currently, there is no direct experimental evidence supporting tau palmitoylation or its direct involvement in tau dysregulation via palmitoylation, but increased palmitate levels are observed to promote tau’s phosphorylation at residue S199/202 and S214; and acetylation at residue K280 (García-Cruz and Arias, 2024; Wlodarczyk et al., 2024). These modifications contribute to tau mislocalization and toxic oligomerization through dysregulated GSK3β and mTOR kinase pathways. Additionally, NAD^+^ dependent Sirtuin 1 (SIRT1, a protective enzyme) activity is observed to be reduced in neurons with increased palmitate levels. The increased palmitate levels tend to disturb the energy metabolism, by shifting from metabolizing glucose to palmitate, which undergoes β-oxidation and generates NADH by utilizing the NAD^+^. The disturbance in NAD^+^ balance reduces the SIRT1 activity, promoting pro-inflammatory stress responses caused due to intracellular ceramide accumulation leading to increased BACE1 expression (Calvo-Ochoa et al., 2017; Flores-León et al., 2019). Few studies reported that tau aggregation is indirectly linked with palmitoylation of its interacting proteins such as fyn (a tyrosine kinase) and amyloid-beta precursor protein (APP). Low levels of tau and fyn are associated with postsynaptic N-methyl-D-aspartate (NMDA) receptors, which are regulated by postsynaptic density protein 95 (PSD-95). PSD-95 is a dense complex scaffolding protein found during neuron excitation, involving in synaptic organization and plasticity. In pathological condition, fyn enhances the hyperphosphorylation of tau, then the tau delivers the fyn kinase to PSD-95 in the post synapse and binds, triggering Aβ excitotoxicity. A hyperphosphorylation of fyn is observed to be involved in causing Aβ excitotoxicity due to a mutation p.T209S in zDHHC21. Similarly, this mutation is also observed to cause Aβ excitotoxicity, when it targets APP (Tezuka et al., 1999; Li et al., 2023; Wlodarczyk et al., 2024). These processes collectively impair microtubule stability and vesicular trafficking, contributing to axonal transport deficits. While tau may not be a direct palmitoylation target, its pathological effects on axonal transport are strongly influenced by palmitoylation-dependent signaling and protein interactions, underscoring the importance of including tau in discussions of palmitoylation in neurodegenerative disease.

#### 2.3.2 MAP1B

Microtubule-associated protein 1B, another significant MAP, is necessary for regulating retrograde mitochondrial transport. By interacting with dynein and kinesin, MAP1B affects the velocity and direction of axonal transport. It ensures proper mitochondrial distribution for maintaining synaptic energy homeostasis and neuron survival. Dysregulation of MAP1B function causes impaired mitochondrial transport, disrupting neuronal energy supply and contributing to axonal degeneration (Jiménez-Mateos et al., 2006; Tortosa et al., 2013). Disruption of MAP1B causes decreased spastin function and thus disrupts the microtubule organization, affecting both anterograde and retrograde movement, leading to progressive motor dysfunction. Spastin is a microtubule-severing enzyme crucial for microtubule remodeling and transport efficiency. It regulates the microtubule’s length and spacing, promoting effective axonal growth and cargo transport. Dysregulation of spastin activity connects with hereditary spastic paraplegia (HSP), a neurodegenerative disorder characterized by axonal degeneration and impaired transport (Jiménez-Mateos et al., 2006).

#### 2.3.3 MAP6

Microtubule-associated protein 6, also known as stable tubule-only polypeptide, is essential for microtubule stabilization in axons. Its function is tightly regulated by palmitoylation, which controls MAP6 shuttling between the membranes and microtubules. Palmitoylation of MAP6 is essential for axonal maturation and microtubule stability, and disruptions in this process are linked to axonal transport defects and neurodegeneration. MAP6 binds to microtubules, ensuring their structural integrity and supporting axon development (Tortosa et al., 2013; Deloulme et al., 2015).

#### 2.3.4 Ankyrin repeats

Ankyrin repeat proteins like Notch, Mask, and NompC are involved in microtubule regulation in Drosophila. The KH-domain of mask protein especially has a negative regulatory effect on microtubule stability, aiding synaptic growth and axonal transport. Stathmin, a microtubule regulator, interacts with mask protein to regulate the efficiency of neuronal transport and stability of the synaptic terminal (Martinez et al., 2021). When mask activity is dysregulated, microtubule organization is disturbed, culminating in synaptic dysfunction in neurodegenerative disorders.

## 3 Palmitoylation in neurons

Post-translational modifications in axonal transport are essential in maintaining neuronal stability and function, for example interaction between tau protein and microtubules are regulated by phosphorylation. PTMs like phosphorylation easily influence tau’s capacity to bind with microtubules, leading to tau aggregation and causing neurodegenerative conditions like AD. Phosphorylation is one of the widely studied modifications; phosphorylation of tau and its significant impact on neuron functioning is the well-known hallmark for the degeneration and instability of neurons. Phosphorylation modifies tau at the microtubule-binding sites and disrupts the stability of microtubules, which is necessary for maintaining neuronal integrity and intracellular transport (Alonso et al., 2018; Fleurima and Holmes, 2020). Beyond phosphorylation, other PTMs, like acetylation, also influence tau behavior. Acetylation at specific sites, such as lysine 280, enhances tau aggregation and compromises microtubule assembly. This modification promotes the formation of toxic tau species while interfering with tau’s interactions with microtubules and its functional part in autophagy (Haj-Yahya and Lashuel, 2018; Esteves et al., 2019). However, acetylation can also modulate phosphorylation, reducing its effects at specific sites and slowing tau fibrillar assembly. This dual role suggests that acetylation may play a compensatory mechanism, potentially counteracting a few detrimental effects of hyperphosphorylation (Cantrelle et al., 2021).

Palmitoylation is the reversible attachment of palmitic acid at the cysteine residue on proteins, which plays an essential role in modulating the localization, stabilization and interaction of the proteins. A family of zDHHC palmitoyl acyltransferases (PATs), catalyzes the palmitoylation modifications, where the enzymatic addition of a 16-carbon palmitic acid to a specific cysteine residue takes place via a thioester linkage. By facilitating the transfer of palmitate to target protein via thioester linkage, the hydrophobicity of the protein increases and promotes the proteins association with the cellular membrane (Dalva, 2009; Ramzan et al., 2023). This lipid modification is reversible. Depalmitoylation, the removal of the palmitate group from the palmitoylated site, is facilitated by acyl-protein thioesterases (APTs), which allows the dynamic regulation of protein function and localization in response to cellular signals.

### 3.1 Palmitoyl acyltransferases (PATs)

The zDHHC family comprises multiple isoforms of PATs, each with distinct substrate specificities and tissue distributions. In neurons, specific zDHHC enzymes have been associated with palmitoylation of key synaptic proteins, thereby modulating the neurotransmission and synaptic plasticity. The zDHHC enzymes localization and activity are tightly regulated, ensuring precise spatial and temporal control over protein palmitoylation states within neuronal circuits. In neurons, the palmitoylation landscape is orchestrated by a repertoire of zDHHC palmitoyl acyltransferases, each exhibiting unique substrate specificities and regulatory mechanisms. PATs are the crucial factor in palmitoylation for regulating protein trafficking, targeting and function. They are established PATs with specificity to involve in certain processes which are discussed below. The following are the list of a few important PATs involved in several neuronal functioning and pathways.

#### 3.1.1 zDHHC5/8

A comprehensive study is conducted on identifying PATs that are specifically involved in enriching the dorsal root ganglion (DRG) axons and identified that zDHHC5 and zDHHC8 are uniquely involved in enriching DRG axons. zDHHC5 and zDHHC8 are also very frequently observed in several neuronal studies, which in the study performed by Collura et al. (2020), specifically facilitates palmitoylation of Gp130 receptor, a membrane glycoprotein receptor involved in axonal retrograde signaling and involved in activating JAK/STAT3 pathways. Palmitoylated Gp130 surface expressions are vital for the activation of JAK/STAT3 pathways. JAK/STAT3 pathway in axonal transport is responsible for transmitting information from the axon to the cell body, a process critical for neuronal development, injury response, and maintenance. This signaling is activated due to extracellular response, when Gp130 is not palmitoylated by zDHHC5/8, their localization to the surface is disrupted, affecting the axonal signaling and transport mechanisms (Collura et al., 2020). Gp130 with zDHHC5 palmitoylation upon successful surface expression, interacts with the neuropoietic cytokines like ciliary neurotrophic factor (CNTF) activating the JAK3, phosphorylating Gp130 and STAT3. When the STAT3 is phosphorylated, they are translocated to neuronal cell body as part of retrograde signaling, which essentially start expressing gene as a response to neuronal development, injury response, and maintenance (Li et al., 2012). Together, these findings suggest a mechanistic link between zDHHC5/8-mediated palmitoylation and retrograde axonal signaling via Gp130, with *in vivo* and cell-based studies supporting a causal role in transport-related signaling disruptions relevant to neurodegenerative disease.

#### 3.1.2 zDHHC2/3

The role of zDHHC2 and zDHHC3 in regulating synaptic function through palmitoylation has been substantiated through genetic knockdown and knockout studies, providing strong mechanistic insights where A-kinase anchoring protein (AKAP)-79/150, a scaffold protein associated with α-amino-3-hydroxy-5-methyl-4-isoxazolepropionic acid (AMPA) receptors is palmitoylated by zDHHC2. Palmitoylation of AKAP79/150 regulates exocytosis, AMPA receptor mediated synaptic potential, post-synaptic recruitment and dendritic spine enlargement by recycling endosomes (Woolfrey et al., 2015). zDHHC2 and zDHHC3 are known to palmitoylate PSD-95, another scaffolding protein involved in neuron synapse essential for synaptic strength and plasticity. PSD-95, a major scaffolding protein essential in excitatory synapses, which is also palmitoylated by zDHHC2. PSD-95 is important for maintaining proper synaptic targeting, stabilizing and trafficking glutamate receptors, particularly AMPA receptors, to ensure that lipid modification of zDHHC2 is necessary. The *in vitro* knockdown study of zDHHC2 shows disruption in palmitoylation of PSD-95, resulting in impaired synaptic clustering and decreased synaptic strength, effects reversible with restoration of zDHHC2 expression. zDHHC2 also contributes to AMPA receptor homeostasis, influencing the receptor recruitment to synaptic membranes and subsequent internalization by regulating the cycle of palmitoylation and depalmitoylation of PSD-95. Within the Golgi apparatus, zDHHC3 mediates the palmitoylation of AMPA receptor sub-units like GluA1 and GluA2. These lipid modifications prevent premature trafficking of receptors to the plasma membrane, thus guaranteeing appropriate receptor maturation. In addition, zDHHC3 palmitoylates the γ2 subunit of GABA_A receptors, a process essential for synaptic clustering and surface stabilization of these inhibitory receptors. The *in vivo* zDHHC3 knockout mice study displays altered palmitoylation of GABA_A receptor γ2 subunits and exhibit deficiencies in inhibitory synapse formation and function (Peng et al., 2024). These mechanistic disruptions directly link zDHHC2/3 dysfunction to impaired excitatory/inhibitory synaptic balance, a key feature of neurodegenerative diseases such as AD and PD, where synaptic dysfunction precedes neuronal loss. The functional consequences of zDHHC2/3 knockdown and knockout in both *in vitro* and *in vivo* models provide strong causal evidence that dysregulation of these enzymes impairs synaptic transport processes, contributing directly to excitatory/inhibitory imbalances observed in neurodegenerative diseases.

#### 3.1.3 zDHHC17

zDHHC17 is localized at the somatic golgi apparatus, where it prepares dual leucine-zipper kinase (DLK) and nicotinamide mononucleotide adenylyltransferase-2 (NMNAT2) for vesicular transport by palmitoylation. These modifications are essential for DLK-mediated cell body degeneration after axonal injury and NMNAT2-dependent maintenance of distal axon integrity in healthy neurons. Retrograde signaling from the axon to the cell body is activated by DLK palmitoylation, which triggers the JNK pathway activation and in response to injury promotes cell body degeneration. Anterograde transport to the distal axon is facilitated to distal axon by NMNAT2 Palmitoylation, which preserves NAD^+^ levels to maintain axonal health. The *in vivo* studies conducted by Niu et al. (2020) plainly demonstrates that zDHHC17 is essential for maintaining axonal health by palmitoylating key substrates such as DLK and NMNAT2. Mouse models lacking zDHHC17 displays DLK and NMNAT2 palmitoylation impairment, which leads to dysfunctional retrograde JNK signaling and accelerated axonal degeneration following neuronal injury. This axonal transport disruption and injury signaling cascades directly involves zDHHC17 dysregulation in neurodegenerative disease mechanisms characterized by axonal loss. Furthermore, the knockdown confirms that zDHHC17 activity is required for proper vesicular trafficking of NMNAT2 to distal axons, a process critical for sustaining NAD^+^ metabolism and axonal survival. Overall, zDHHC17 has been causally implicated in axonal transport maintenance and injury response through its palmitoylation of DLK and NMNAT2, with genetic knockout models clearly demonstrating its essential role in preventing axonal degeneration and supporting neuroprotective transport mechanisms.

Despite strong evidence supporting individual zDHHC enzyme roles in axonal transport and synaptic function, it is crucial to differentiate well-validated causal pathways from correlative observations. To provide a consolidated overview, the following Table 1 summarizes key palmitoylated proteins, their regulatory enzymes, and the known implications for axonal trafficking and disease associations. This context sets the foundation for the subsequent section, which explores how palmitoylation as a dynamic post-translational modification orchestrates axonal cargo localization, vesicular transport, and long-range neuronal connectivity under both physiological and pathological conditions.

**TABLE 1:** Key palmitoylated proteins, their regulatory enzymes, and roles in axonal transport and neurological diseases.

Proteins	Associated enzymes	Function in axonal transport	Associated disease	References
APP	zDHHC 3, 4, 7, 15, 20, 21	Responsible for the palmitoylation of β−site amyloid precursor protein shear enzyme 1	AD	Cho and Park, 2016; Qian et al., 2025
PSD-95	zDHHC 2, 3, 15	Scaffolds post-synaptic receptors; regulates AMPA receptor clustering at synapses	AD, Schizophrenia	Cho and Park, 2016; Qian et al., 2025
SNCA	zDHHC9	Pathological aggregation in Parkinson’s disease	PD	Qian et al., 2025
MAP-6	zDHHC 2, 3, 6, 7, 15, 20	Associate with the golgi apparatus through palmitoylation of their N-terminal domains	PD	Gory-Fauré et al., 2014; Tortosa et al., 2017; Qian et al., 2025
HTT	zDHHC17 (HIP14), zDHHC13	Mutant protein aggregation in Huntington’s disease (HD)	HD	Huang et al., 2004; Holland and Thomas, 2017; Qian et al., 2025
NMNAT2	zDHHC 7, 17	Axon survival factor; palmitoylation enables vesicle association for anterograde transport	ALS	Milde and Coleman, 2014; Holland and Thomas, 2017; Summers et al., 2018
GAD65	zDHHC 3, 7, 9, 17	GABA synthesis; palmitoylation ensures axonal targeting	HD, schizophrenia, epilepsy	Rush et al., 2012; Cho and Park, 2016; De and Sadhukhan, 2018
SNAP-25	zDHHC 3, 7, 13, 17	Mediates synaptic vesicle fusion; anterograde transport of vesicles	AD, HD, cognitive disorders	Greaves et al., 2010; Cho and Park, 2016
Ankyrin-G	zDHHC 5, 8	Targeting to membranes and axon initial segment	Neurodegeneration, bipolar disorders, and schizophrenia	He et al., 2012, 2014; Chagula et al., 2020
DLK	zDHHC10, 11, 22, 23	Localization to vesicles, also necessary for protein-protein interactions and kinase activity.	AD, PD, ALS	Holland et al., 2016; Holland and Thomas, 2017; Bu et al., 2023

## 4 Role of palmitoylation in axonal transport

Palmitoylation plays a fundamental role in maintaining the efficiency and directionality of axonal transport in healthy neurons. S-palmitoylation is a reversible lipid modification that dynamically regulates protein localization, membrane association, and intracellular trafficking, all of which are crucial for neuronal polarity and function. Palmitoylation influences the recruitment and activation of motor proteins such as kinesin by altering the availability and placement of scaffolding and adaptor proteins, which regulates recruitment and activation process. The regulatory layer plays a crucial role in maintaining the directional movement of organelles and vesicles along axonal microtubules (Huang and El-Husseini, 2005; Ernst et al., 2018). The establishment of axonal identity and polarity relies heavily on the localized distribution of specific proteins within specific spatial areas. Palmitoylation aids in this process by directing specific proteins to the axonal compartment, thereby facilitating the organization of microtubules, the transport of cargo, and the maturation that is specific to a particular compartment. The selective protein retention is maintained by a balance between palmitoylation and depalmitoylation, which prevents proteins from being misdirected to dendrites and ensures the continued separation of axonal and dendritic functions (Ernst et al., 2018; Tortosa and Hoogenraad, 2018).Palmitoylation serves as a molecular sorting signal within the secretory pathway, where palmitoylation at the golgi apparatus facilitates the anterograde targeting of cargo, directing proteins into vesicles that will eventually form at the axonal membranes. The process is initiated by the increased number of palmitoylated proteins in the curved regions of the golgi apparatus, promoting vesicle packaging and targeted delivery to specific compartments (Ernst et al., 2018). In addition, cycles of palmitoylation and depalmitoylation enable the transient association of soluble cytoplasmic proteins with golgi-derived vesicles, facilitating efficient and flexible transport within neurons. Depalmitoylation at distal sites, including the ends of axons, enables the selective retention of these proteins in relevant compartments involved in polarized trafficking. The correct localization of several key synaptic proteins requires palmitoylation. Proteins like SNAP25 and GAP43, which are involved in the release and targeting of synaptic vesicles, need to be palmitoylated in order to associate with the membrane and dock the vesicles, thus supporting the development of synapses and neurotransmission processes (Huang and El-Husseini, 2005; Tortosa and Hoogenraad, 2018).

Beyond palmitoylations role in membrane association, they also affect the dynamics of microtubule such as stability, which is vital for maintaining neuronal network and facilitating cargo transportation. MAPs like tau, when subjected to PTMs enhance tau’s association by modulating their binding to microtubule. On contrary, aberrant PTMs of tau is observed causing tau’s to mislocalize and form aggregates followed by impairing microtubule stability and suspected to disrupting axonal transport in AD (García-Cruz and Arias, 2024). Furthermore, palmitoylation of motor proteins, including kinesins and dyneins, influences their interaction with microtubules and cargoes. This modification can regulate the motor proteins activity, affecting their efficiency and directionality of axonal transport. In HD, the motor adaptor protein huntingtin-associated protein 1 (HAP1) palmitoylation modulates its binding to kinesin, thereby influencing the organelles and vesicles transport along microtubules (Keryer et al., 2011). The reversible nature of palmitoylation allows for dynamic regulation of protein localization in response to neuronal activity and signaling events. Depalmitoylation can release proteins from membranes, facilitating their redistribution within the cell or degradation. This cyclical process is essential for the proper synaptic proteins functioning, ion channels, and signaling molecules, ensuring rapid and adaptable responses to changing neuronal conditions (Butland et al., 2014; Zhang et al., 2018).

## 5 Role of dynamic regulation of palmitoylation in aging and neurodegeneration

Palmitoylation is a reversible lipid modification that regulates neuronal plasticity and repair by modulating protein localization and function. This dynamic process is critical for neurotransmitter receptor trafficking, such as AMPA and NMDA receptors, which depend on palmitoylation for surface stability and synaptic signaling (Naumenko and Ponimaskin, 2018). Changes in palmitoylation affect synaptic strength, learning, and memory (Globa and Bamji, 2017), and also influence neuronal excitability by modulating ion channels such as NaV1.6 and NaV1.7 (Pan et al., 2020). Restoring and enhancing palmitoylation of key synaptic proteins can aid aging neurons in synaptic maintenance and cognitive function, and this emerging strategy can be a best counteract to age-related neuronal dysfunctions (Fukata and Fukata, 2010). Correcting palmitoylation-dependent trafficking defects may also prevent synaptic loss and neurodegeneration, particularly in aging neurons where mislocalized proteins contribute to synaptic failure (Ramzan et al., 2023).

Age-related impairments in palmitoylation reduce the function of key receptors like AMPA and NMDA (Zarêba-Kozioł et al., 2021) and cause accumulation of improperly localized proteins due to inefficient depalmitoylation (Koster et al., 2023). Age-related modifications in palmitoylation, which regulates kinase localization activity and affects cellular signaling pathways, are crucial for neuronal survival and lead to neuroprotection impairment, abnormalities in neural signaling and kinase function (Montersino and Thomas, 2015). Additionally, peroxiredoxin-6 (PRDX6) induces oxidative stress and metabolic imbalances as a consequence of defective palmitoylation, which contributes to age-related neurodegeneration (Cao et al., 2022). Age-related changes in lipid modifications result in reduced synaptic efficiency and neurotransmission impairment, also promoting neurodegeneration. Targeting these pathways offers therapeutic potential for neuroprotection in aging.

**FIGURE 2:**
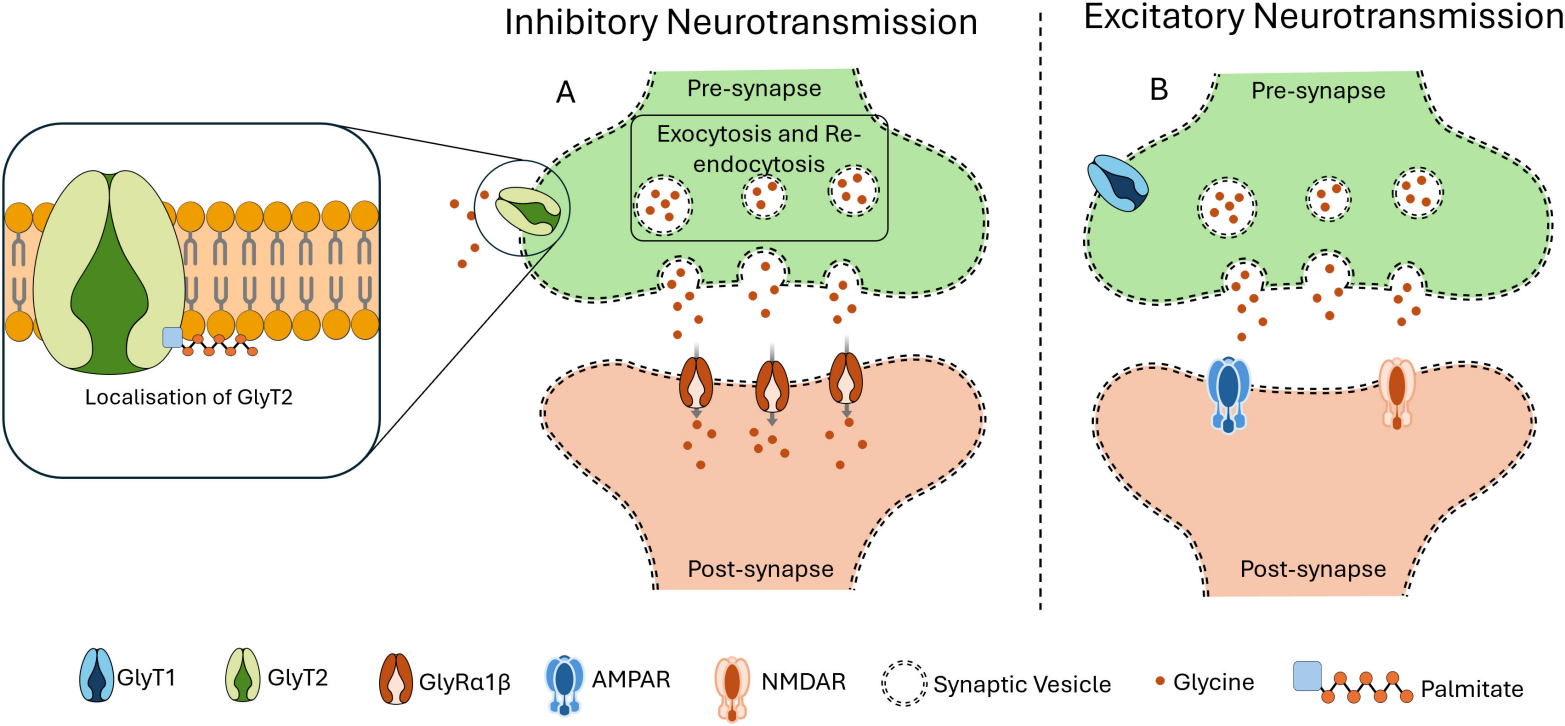
Importance of Palmitoylation in localization. Palmitoylation is an important PTM required for the proper functioning of GlyT2, which is responsible for the reuptake of glycine from the synaptic cleft into the presynaptic terminal. This transporter facilitates the transport of glycine within synaptic vesicles, thereby refilling the intracellular glycine reservoir necessary for inhibitory neurotransmission mechanism. Once internalized, these vesicles are refilled with glycine, preparing them for subsequent release in response to an action potential. As illustrated in panel **(A)**, GlyT2 ensures the efficient uptake of glycine at the presynaptic site and its replenishment into synaptic vesicles. Upon action potential triggers the glycine is released, then the glycine receptors (GlyRα1β) on the postsynaptic membrane uptake the glycine to exert its inhibitory neurotransmission effect. This tightly regulated mechanism depends on the accurate localization of GlyT2 to the presynaptic membrane. However, as shown in panel **(B)**, when palmitoylation of GlyT2 is disrupted or inhibited, its presynaptic localization is impaired. This leads to a reduction or loss of GlyT2 at the synapse, compromising glycine refilling into vesicles and causing excitatory neurotransmission.

Several structural and functional changes contribute to cognitive decline and reduced plasticity with aging neurons. Palmitoylation is one of the important factors in maintaining neuronal function during aging, as it is used to sustain intracellular transport mechanisms. But during aging the efficiency of intracellular transport tends to reduce, impairing synaptic transmissions and leads to neurodegeneration. For example, palmitoylation of dopamine transporter (DAT) regulates trafficking and degradation signaling that are essential for maintaining motor and cognitive functioning. Dysregulated palmitoylation of DATs is associated with age-related cognitive decline by impairing the neurotransmitter balance (Zamzow et al., 2019). By palmitoylation of glycine transporters (GlyT2) in lipid rafts, GlyT2 is localized and preserves the synaptic vesicle transport, mechanism illustrated in Figure 2. Localization of GlyT2 is important for proper neurotransmitter recycling. Inhibitory neurotransmission is disrupted causing reduced synaptic stability and increased neuronal excitability due to age-related defects in palmitoylation (Felipe et al., 2024). Palmitoylation also ensures proper membrane localization and functioning of voltage-gated sodium channels (NaV1.7) by regulating ion channel activity. Sensory perceptions like pain can be affected by altering neuronal excitability when palmitoylation activity is reduced with aging (Tang et al., 2024). Palmitoylation also supports dendritic transport by modifying scaffolding proteins like ankyrin-B, which are required to localize sodium channels in dendrites and maintain synaptic excitability. Loss of palmitoylation in these proteins during aging impairs synaptic transmission and cognitive function (Gupta and Jenkins, 2023).

## 6 Role of palmitoylation in neurodegenerative diseases

**FIGURE 3:**
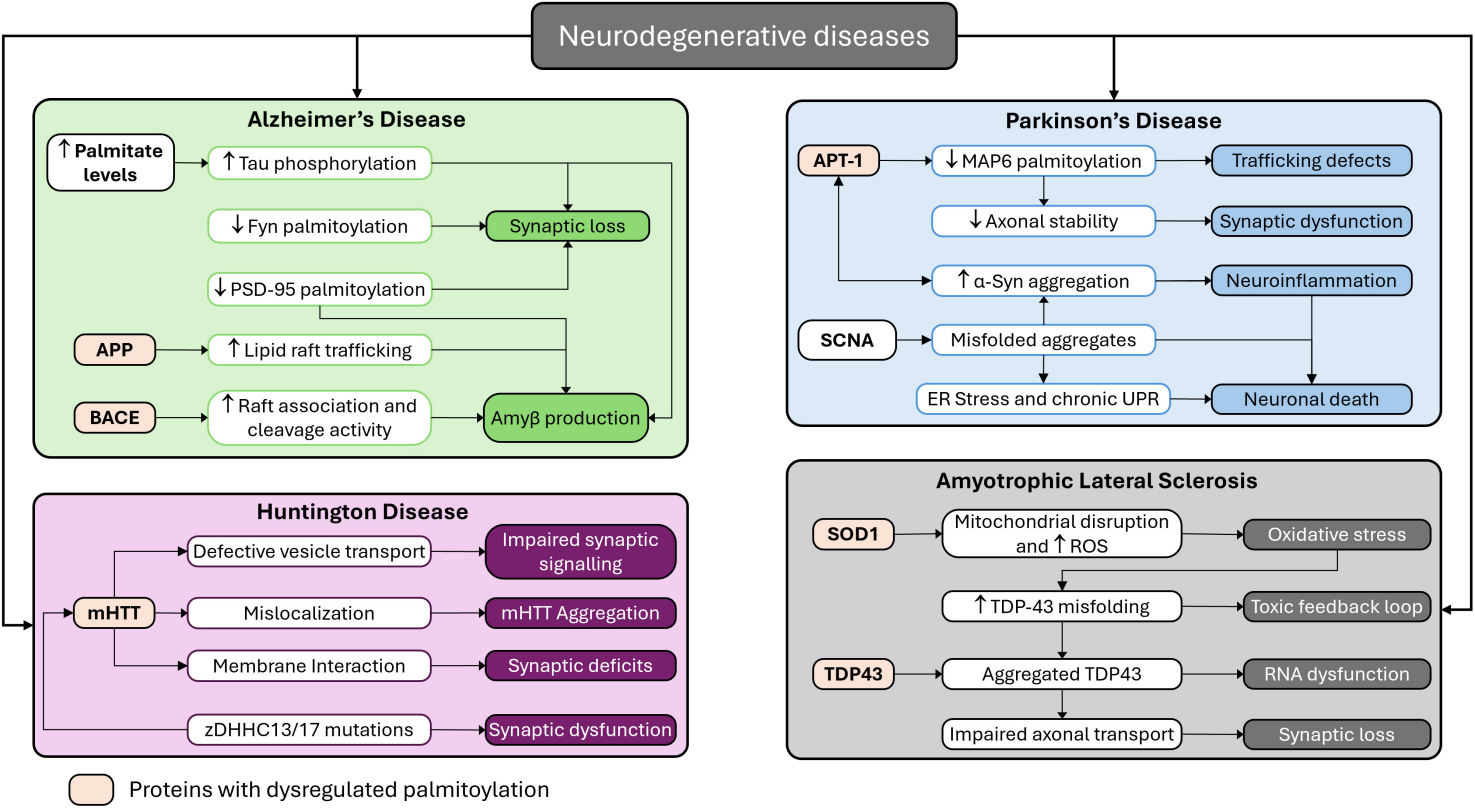
Mapping palmitoylation dysregulation across major neurodegenerative diseases. This schematic illustrates the role of palmitoylation dysregulation in the pathogenesis of four major neurodegenerative disorders: AD, PD, ALS, and HD.

Dysregulation in palmitoylation can interfere with protein localization, stability, and interactions, affecting several neuronal functions leading to pathogenesis. Dysregulation of palmitoylation is linked with several neurodegenerative diseases that can cause AD, PD, ALS and more. A schematic diagram is mentioned in Figure 3 mentioning the mapping of palmitoylation dysregulation across major neurodegenerative diseases. In AD, palmitoylation of APP enhances its trafficking to lipid rafts and mitochondria-associated membranes, where BACE1 cleaves it to form neurotoxic Aβ peptides. Palmitoylation of BACE1 itself stabilizes its raft association and enzymatic activity, amplifying Aβ generation. Additionally, exposure to Aβ induces depalmitoylation of PSD-95, leading to synaptic deterioration. Palmitoylation of fyn kinase is required for its synaptic localization, and its loss contributes to tau hyperphosphorylation and synaptic toxicity. Elevated palmitate levels also indirectly drive tau mislocalization via kinase activation and phosphatase disruption (Bhattacharyya et al., 2013; Li et al., 2023; Wlodarczyk et al., 2024). In PD, although α-syn is not directly palmitoylated, its aggregation is modulated by the depalmitoylating enzyme depalmitoylase acyl-protein-thioesterase-1 (APT1). APT1 overactivity increases α-syn phosphorylation at serine-129, promoting cytoplasmic inclusions. APT1 also depalmitoylates MAP6, a microtubule-associated protein necessary for vesicle trafficking and neuronal integrity. Inhibition of APT1 restores MAP6 palmitoylation and reduces α-syn aggregation, highlighting its therapeutic potential (Ho et al., 2021). In HD, Wild-type huntingtin (HTT) is palmitoylated by ZDHHC17 and ZDHHC13, which stabilizes its membrane interactions and intracellular trafficking. In the mutant form (mHTT), reduced palmitoylation contributes to its aggregation and mislocalization, exacerbating neuronal dysfunction. Mutations in ZDHHC17/13 impair synaptic function and accelerate disease progression (Lemarié et al., 2023). In ALS, Mutant superoxide dismutase 1 (SOD1) undergoes palmitoylation that enhances its association with mitochondrial membranes, inducing oxidative stress and neuronal death. Similarly, defective palmitoylation of TDP-43 results in its cytoplasmic mislocalization and aggregation, impairing RNA processing and contributing to neurotoxicity (Ramzan et al., 2023). These findings emphasize the central role of palmitoylation in regulating protein localization and function in neurodegeneration. Targeting palmitoylating and depalmitoylating enzymes, such as APT1 and zDHHCs offers promising avenues for therapeutic intervention.

### 6.1 Palmitoylation in Alzheimer’s disease

In AD, interaction between tau protein and Aβ peptides is one of the major players in triggering synaptic dysfunction and neuronal degeneration particularly by disrupting the neuronal transport mechanisms. Tau is one of the largely involved MAPs that helps in facilitating microtubule stability and axonal transport. While direct palmitoylation of tau has not been established, emerging evidence suggests that palmitoylation-related pathways indirectly influence tau phosphorylation, aggregation, and its interactions with key proteins involved in axonal transport and synaptic toxicity. The imbalance between tau 3R and 4R isoform populations can impair the transport of APP, where the 3R isoform dominance favors anterograde movement and 4R isoform dominance favors the retrograde movement in axonal transport. This imbalance between anterograde and retrograde transport leads to disruption of APP dynamics, causing neurodegeneration (Lacovich et al., 2017). More importantly, tau is essential for Aβ-induced neurotoxicity. Suppose tau-expressing neurons degenerate in the presence of Aβ while tau-depleted neurons exhibit resistance. In that case, it is evident that tau plays a critical role in the disruption and neurodegeneration of Aβ-mediated transport (Vossel et al., 2015). Tau hyperphosphorylation driven by Aβ oligomers can disrupt microtubules, causing degeneration of neurons. Due to which, tau’s function in regulating axonal transport can be directly impacted by Aβ, leading to synaptic dysfunction and neurodegeneration (Decker et al., 2010). Furthermore, tau pathology is primarily caused by the ratio of Aβ42/40. Elevated aggregation and tau deposition are linked with an increased Aβ42/40 ratio, which further impairs neuronal transport and builds up to the pathophysiology of AD (Salvadores et al., 2020). These studies collectively indicate that tau and Aβ play a crucial role in disrupting transport mechanisms in AD by the involvement of Aβ oligomer interactions and imbalance in tau isoform impairing axonal transport and synaptic function mechanisms.

Palmitoylation at the cysteine residue is a crucial PTM controlling synaptic protein localization, stability and functionality. Dysregulated palmitoylation causes neurodegeneration due to mutations in zDHHC enzymes and appears to have significant effects on synaptic proteins. For example, flotillin-2, an important synaptic protein that maintains synaptic integrity, is not palmitoylated due to the zDHHC5 enzyme mutation disrupting neuron development and synaptic plasticity (Li et al., 2012). zDHHC2 enzyme mutations disrupt the palmitoylation of AKAP-79/150, important for maintaining synaptic potential and AMPA receptor trafficking. Studies imply that palmitoylation of AKAP-79/150 is important for maintaining synaptic plasticity, where knockdown of zDHHC2 enzymes resulted in impairing AMPA receptor exocytosis, dendritic spine expansion and synaptic strengthening (Woolfrey et al., 2015). GABAergic transmissions are affected by the disrupted palmitoylation of the GABA_A receptor due to a mutation in the zDHHC3 enzyme, also called golgi-specific DHHC zinc finger protein (GODZ). GABAergic transmissions are affected by GODZ enzyme mutation, which impacts inhibitory synapse formation and function (Kilpatrick et al., 2016). δ-catenin palmitoylation is essential for enhancing spine stability, receptor recruitment and synaptic cadherin interactions. Disruption in this process impacts the synaptic plasticity and adhesion with cadherins, severely disturbing memory formation (Brigidi et al., 2014). The interaction between protein interacting with C kinase 1 (PICK1) and zDHHC8 palmitoylates is necessary for cerebellar long-term synaptic depression (LTD), which is disrupted due to loss of zDHHC8 results in impaired LTD and synaptic functions (Thomas et al., 2013). The overall stability and function of the DATs are effectively maintained by zDHHC enzymes such as zDHHC2, zDHHC3, zDHHC8, zDHHC15, and zDHHC17. Defects in these zDHHC enzymes can induce disruptions in the dopaminergic system, causing neurodegenerative disorders (Rastedt et al., 2014). The above finding exhibits that mutations in zDHHC enzymes can affect the role of essential synaptic protein palmitoylation, which consequently impairs synaptic plasticity and neurotransmission and increases the chances of neurodegenerative diseases. Studying the effects of zDHHC mutations on the palmitoylation of synaptic proteins could provide essential knowledge on the molecular mechanisms behind neuronal degeneration and identify potential opportunities for intervention.

### 6.2 Palmitoylation in Parkinson’s disease

In PD, α-synuclein, a presynaptic neuronal protein, impairs mitochondrial transport and function when pathologically aggregated. These aggregations cause morphological disruptions in mitochondria by fragmentation and swelling, which, in process, damages the membrane potential of mitochondria and results in neuronal apoptosis. It is due to the subtle interaction of α-synuclein with mitochondrial proteins involved in the mitochondrial permeability transition pore, which disturbs the energy production and homeostasis in mitochondria (Lurette et al., 2023). Additionally, the dynamics of mitochondria are adversely affected by α-synuclein aggregation, where the balance between the fission and fusion process is impaired, causing mitochondrial fragmentation. This disruption changes the transport of mitochondria in the axonal transport mechanism, triggering oxidative stress and energy deficits within neurons (Pozo Devoto and Falzone, 2017). Additionally, translocase of the outer membrane 40 (TOM40), a mitochondrial protein involved in import mechanisms, was observed to be reduced by aggregated α-synuclein. Downregulation of TOM40 impairs the import of necessary proteins across mitochondria, increasing oxidative stress, decreasing energy levels, and compromising neuron viability (Vasquez et al., 2024). Aggregation of α-synuclein also impairs the microtubule-dependent transport mechanisms, which mislocalizes the mitochondria in neurons, causing severe energy depletion. Energy depletion due to mislocalized mitochondria occurs precisely at the synaptic terminal, contributing to neurodegeneration in PD (Zaltieri et al., 2015). Palmitoylation plays an important role in vesicle trafficking and synaptic activity regulation and increases the hydrophobicity of the proteins, thus accelerating their association with cellular membranes and affecting their localization and function (Ji and Skup, 2021).

Palmitoylation, in synaptic vesicle trafficking, regulates the synaptic protein involved in neurotransmitter release, such as the Soluble N-ethylmaleimide-sensitive factor attachment protein receptor (SNARE) complex. For example, Synaptosomal-associated protein of 25 kDa (SNAP25), a component from the SNARE complex, is required for proper localization of the SNARE complex toward the plasma membrane. Disruption in palmitoylation of SNAP25 impacts the synaptic vesicle exocytosis and impairs synaptic transmission and neurotransmitter release (Peng et al., 2024). Furthermore, palmitoylation impacts synaptic strength and plasticity by modulating the clustering and function of the postsynaptic receptor. For instance, the trafficking and surface expression of AMPA-type glutamate receptors is affected by palmitoylation, which is vital for the synaptic plasticity activities that represent learning and memory (Sohn and Park, 2019). Interestingly, changes in palmitoylation have been linked with PD pathogenesis. Focusing on the palmitoylation pathways may provide the potential for therapy in addressing α-synuclein-related neurodegeneration (Zarêba-Kozioł et al., 2021). The aggregation of α-synuclein in PD disrupts mitochondrial transport and function through multiple mechanisms through several processes, such as mitochondrial impairment, interfering with mitochondrial dynamics, reduction in protein import in mitochondria and disrupting microtubule-based transport. This proves that palmitoylation serves as a significant regulatory mechanism in vesicle trafficking and synaptic function, which, when dysregulated, causes synaptic deficits in PD.

## 7 Discussion

Palmitoylation is a reversible, lipid modification involving the attachment of palmitic acid to specific cysteine residues on proteins, which plays an essential role in modulating the localization, stabilization and interaction of the proteins. Emerging research highlights the therapeutic potential of reducing the progression of neurodegenerative disease by targeting the palmitoylation processes. The aim of targeting palmitoylation as therapeutic strategies is to rectify the lipid modification imbalance to restore or preserve the neuronal function. Regulation of palmitoylating and depalmitoylating agents like PATs, APTs and PPTs are explored to check their potential to rectify the aberrant lipid modifications in proteins associated with neurodegeneration (Hornemann, 2015). Additionally, in ischemic stroke models, palmitoylation-dependent pathways like JNK3 are targeted by neuropeptides shows promising neuroprotective effects. Along with this, the pharmacological modulation of zDHHC2 is studied in restoring synaptic functions in neurodegenerative diseases (Liao et al., 2024). Gene therapy and RNA-based interventions are also some promising approaches, that mainly targets the miRNAs involved in palmitoylation-related neuronal functions, offering potential treatments for diabetic neuropathy and sensory neurodegeneration (Zochodne et al., 2016). Despite encouraging preclinical findings, no palmitoylation-targeting therapy has yet progressed to advanced clinical trials for neurodegenerative diseases, highlighting the early stage of translational development in this field.

One promising strategy involves modulating the activity of PATs, particularly the zDHHC family of enzymes which catalyzes palmitoylation process. For instance, zDHHC5 and zDHHC8 are uniquely observed in DRG axons, where they regulate the retrograde signaling via the Gp130/JAK/STAT3 pathway (Collura et al., 2020). Axonal signal transmission is impaired when the enzymes zDHHC5 and zDHHC8 are inhibited, suggesting which upon activation can reduce the transport defects in neurodegenerative conditions, by enhancing axonal integrity. Additionally, zDHHC5 also catalyzes GRIP1, targeting it to dendritic endosomes to regulate AMPA receptor recycling, a process crucial for synaptic function and cognitive processes (Thomas et al., 2012). Similarly, PSD-95 is catalyzed by zDHHC2 localization to dendritic vesicles, enhancing zDHHC2 activity may strengthen the synaptic plasticity, which is often compromised in neurodegenerative diseases (Greaves et al., 2011).

### 7.1 Therapeutics and current challenges

The clinical application of palmitoylation-targeted therapies is obstructed by several challenges, despite many promising directions. Many palmitoylating inhibitors like 2-bromopalmitate (2-BP) targets several zDHHC enzymes, which may have neurotoxic effects by interacting with off-target zDHHC enzymes (Chaturvedi et al., 2024). A study conducted by Bai et al. (2024) studied the use of proteolysis-targeting chimeras (PROTACs) to target the membrane bound zDHHC and PAT interactions, and succeeded in degradation of the zDHHC5 and zDHHC20 enzymes and along with reduction in palmitoylation of their substrate. Additionally, the palmitoylation process on the substrate are observed to substituted by other non-targeted zDHHCs. This study in overall demonstrated the feasibility of targeting the zDHHC and PATs interactions, but with the limited enzyme redundancy where other non-targeted zDHHCs are observed to be compensating the loss of targeted zDHHCs. This functional redundancy among zDHHC enzymes complicates targeted therapeutic strategies, as inhibition of one isoform may be compensated by others, limiting long-term efficacy unless combinatorial or context-specific approaches are developed. Many zDHHC enzymes are complicated to target due to insufficient knowledge on the substrate specificity and regulatory mechanisms.

Palmitoylation inhibitors, such as 2-BP, cerulenin, tunicamycin and cyano-myracrylamide (CMA) targets almost all palmitoylation modification, but they lack isoform specificity and broadly inhibit the entire zDHHC family of palmitoyl PATs as well as other unrelated proteins, leading to widespread off-target effects and toxicity (Jennings et al., 2009; Azizi et al., 2021; Yu et al., 2024). The catalytic domains of DHHC enzymes are highly conserved, making it technically challenging to design isoform or substrate-specific inhibitors (De and Sadhukhan, 2018). Natural flavonoids such as lutein, 5-hydroxyflavone, and 6-hydroxyflavone have been identified based on *in silico* screening as selective inhibitors of zDHHC20-mediated palmitoylation, represents a different therapeutic approach with potentially reduced adverse consequences (Chaturvedi et al., 2024). Several researchers are actively involved in developing palmitoylation inhibitors or activators as a neuroprotective strategy. For example, 2-BP and tunicamycin, a zDHHC inhibitor have been explored to check for their potential in mitigating the pathological palmitoylation in neurodegenerative diseases, but their lack of specificity and unintended cellular effects along with cytotoxic effects sets the limitations (Pei and Piao, 2024).

Lipid-based therapeutics provides additional avenue for modulating protein localization and function through palmitoylation. But, lipid-based molecules often exhibit poor bioavailability, necessitating the development of novel delivery systems to ensure effective therapeutic concentrations in neuronal tissues. A study on nitro-fatty acids (a lipid based therapy) by Hansen et al. (2018) observed that covalent modification of signaling protein stimulator of IFN genes (STING) via nitro-alkylation inhibits the palmitoylation and reduces the STING mediated signaling, a signaling pathway when triggered excessively leads to uncontrolled inflammation and tissue damage in inflammatory and autoimmune disease. Since the nitro-fatty acids utilized in Hansel et al. study is formed in response to viral infection, heir long-term safety, efficacy, and potential side effects remain unclear. Given the conditions, dysregulated palmitoylation modifications on specific zDHHC enzymes likes zDHHC9, zDHHC13, and zDHHC21 are targeted in AD, HD, schizophrenia, and glioma, suggesting as a therapeutic approach (Liao et al., 2024). Moreover, modulating palmitoyl-protein thioesterases (PPTs), which are responsible for depalmitoylation, could restore balance in protein palmitoylation, preventing the toxic accumulation of misfolded proteins characteristic of neurodegenerative disorders. There is a lack of robust clinical data demonstrating efficacy or safety in humans, and the blood-brain barrier remains a significant obstacle for CNS-targeted delivery (Wlodarczyk et al., 2024). The major challenge in developing lipid-based inhibitors is the poor solubility of small molecules. However, recent advances in the liposomal transport delivery of hydrophobic medications could offer a potential solution.

### 7.2 Future direction

This review consolidates current mechanistic understanding of palmitoylation’s role in modulating microtubules, motor proteins, and microtubule-associated proteins, emphasizing their dysregulation in Alzheimer’s, Parkinson’s, and ALS. It critically appraises therapeutic opportunities to selectively target palmitoylation enzymes, acknowledges challenges such as inhibitor specificity and delivery, and underscores promising research directions including gene therapy, RNA-based modulation, and lipid nanoparticle delivery systems. Future research could explore the development of isoform- and substrate-specific modulators by targeting unique N- or C-terminal regions of DHHC enzymes or leveraging structural distinctions identified in recent crystallography studies. Additionally, investigating advanced delivery systems, such as nanoparticles or viral vector-based approaches may enhance brain bioavailability while minimizing peripheral toxicity and systemic exposure. Another promising avenue could involve identifying palmitoylation-specific biomarkers (e.g., through S-acylation profiling) to monitor target engagement in clinical trials. Advancements in acyl-biotin exchange assays and mass spectrometry-based S-palmitoylome profiling may enable the identification of palmitoylation-specific biomarkers, offering tools for both diagnosis and therapeutic monitoring (Zhao et al., 2010). Integrating palmitoylation modulation into neurodegeneration treatments requires multifaceted strategies, such as combining palmitoylation modulators with existing neuroprotective drugs could enhance synaptic stability and function in diseases like AD and PD. Personalized medicine approaches, involving the identification of patient-specific zDHHC mutations, could enable tailored treatments for various neurodegenerative disorders. Overall, while the therapeutic modulation of palmitoylation holds significant promise, realizing its clinical impact will require overcoming technical, biological, and regulatory barriers through collaborative, interdisciplinary research.

## 8 Conclusion

Axonal transport is an intricate and fundamental process that ensures neuronal function and survival, which is on several cases regulated by key roles of palmitoylation modifications. Impaired axonal transport due to disruptions in palmitoylation modifications is widely associated with neurodegenerative disease pathology like AD, PD, HD, and ALS. Maintaining proper palmitoylation balance is therefore important for preserving axonal transport efficiency and overall neuronal health. Targeting palmitoylation, particularly through modulation of zDHHC enzymes and associated pathways, holds promise for restoring synaptic integrity and neuronal health. Although pharmacological and genetic strategies, such as zDHHC inhibitors, RNA-based interventions, and lipid-based therapeutics, have shown potential, challenges remain. These include the lack of enzyme isoform specificity, off-target effects, and delivery barriers across the blood-brain barrier. Future research focus on identifying palmitoylation dynamics in neuronal axonal transport elucidating the precise signaling pathways and finding specific palmitoylating and depalmitoylating enzymes regulating the signaling pathway could paves way for promising avenues for altering or possibly slowing the disease condition. A deeper understanding of the interplay between palmitoylation and neuronal transport will not only enhance our knowledge of fundamental neurobiology but also pave way for innovative strategies to combat neurodegeneration. By bringing together recent findings on the regulation of axonal transport machinery by palmitoylation and its dysregulation in major neurodegenerative diseases, this review fills an important gap in the current literature.
